# Functional Connectivity and Genetic Profile of a “Double-Cortex”-Like Malformation

**DOI:** 10.3389/fnint.2018.00022

**Published:** 2018-06-12

**Authors:** Giulia Sprugnoli, Giampaolo Vatti, Simone Rossi, Alfonso Cerase, Alessandra Renieri, Maria A. Mencarelli, Federico Zara, Alessandro Rossi, Emiliano Santarnecchi

**Affiliations:** ^1^Department of Medicine, Surgery and Neuroscience, Brain Investigation & Neuromodulation Laboratory, University of Siena, Siena, Italy; ^2^Department of Medicine, Surgery and Neuroscience, Neurology and Clinical Neurophysiology Section, University of Siena, Siena, Italy; ^3^Department of Medicine, Surgery and Neuroscience, Section of Neuroradiology, University of Siena, Siena, Italy; ^4^Department of Medicine, Surgery and Neuroscience, Section of Medical Genetics, University of Siena, Siena, Italy; ^5^Laboratory of Neurogenetics and Neuroscience, Istituto Giannina Gaslini, Genoa, Italy; ^6^Department of Cognitive Neurology, Beth Israel Deaconess Medical Center, Berenson-Allen Center for Noninvasive Brain Stimulation, Harvard Medical School, Boston, MA, United States

**Keywords:** functional connectivity, double cortex syndrome, fMRI, genetic profile, subcortical band heterotopia, double-cortex-like, resting-state

## Abstract

Laminar heterotopia is a rare condition consisting in an extra layer of gray matter under properly migrated cortex; it configures an atypical presentation of periventricular nodular heterotopia (PNH) or a double cortex (DC) syndrome. We conducted an original functional MRI (fMRI) analysis in a drug-resistant epilepsy patient with “double-cortex”-like malformation to reveal her functional connectivity (FC) as well as a wide genetic analysis to identify possible genetic substrates. Heterotopias were segmented into region of interests (ROIs), whose voxel-wise FC was compared to that of (i) its normally migrated counterpart, (ii) its contralateral homologous, and (iii) those of 30 age-matched healthy controls. Extensive genetic analysis was conducted to screen cortical malformations-associated genes. Compared to healthy controls, both laminar heterotopias and the overlying cortex showed significant reduction of FC with the contralateral hemisphere. Two heterozygous variants of uncertain clinical significance were found, involving autosomal recessive disease-causing genes, FAT4 and COL18A1. This first FC analysis of a unique case of “double-cortex”-like malformation revealed a hemispheric connectivity segregation both in the laminar cortex as in the correctly migrated one, with a new pattern of genes’ mutations. Our study suggests the altered FC could have an electrophysiological and functional impact on large-scale brain networks, and the involvement of not yet identified genes in “double-cortex”-like malformation with a possible role of rare variants in recessive genes as pathogenic cofactors.

## Introduction

### Patient’s Data

A 17-year-old right-handed woman referred to our Epilepsy center for a second opinion about a drug-resistant epilepsy, with sub-optimal pharmacological seizures’ control. In fact, multiple daily seizures were reported, specifically generalized tonic-clonic seizures (occurring since the age of nine), simple motor partial seizures (head-turning without aura, since 13 years old) and atonic seizures involving upper arms and neck muscles (since the age of 15). A retardation in the acquisition of language abilities emerged from the interview with the mother (however, no clinical documents were available of this), matching with the impaired language abilities at the time of the evaluation (see **Table 1**). Familial history revealed that the mother was affected by Neurofibromatosis 1, Systemic Lupus Erythematosus and had a normal electroencephalography pattern. A paternal cousin was affected by a frontal cortical dysplasia with mental retardation. The patient showed a complete normal neurological examination. Pharmacological anamnesis revealed that Primidone, Levetiracetam, Lacosamide and Zonisamide have no effects on the patient seizures’ control and actual therapy consisted on Topiramate 150 mg twice daily, Clonazepam 1 mg three times/daily and Oxcarbazepine 900 mg twice daily. Extensive battery of neuropsychological tests revealed moderate mental retardation (V.I.Q. = 57, P.I.Q. = 52, T.I.Q. = 51, Wechsler Intelligence Scale for Children-Revised; Orsini, 1993) as a global impairment of cognitive abilities [sustained and divided attention: Trail making test (TMT) form A and attentional matrices, Corrigan and Hinkeldey, 1987; verbal short-term memory: single digit span and visuo-spatial short-term memory: Corsi visuo-spatial span, Schoenberg et al., 2006; visuo-spatial long-term memory: Rey–Osterrieth complex figure (ROCF), Osterrieth, 1944; language: oral comprehension, Token test, De Renzi and Vignolo, 1962; denomination: Boston Naming Test, Kaplan et al., 1983; non-verbal fluid intelligence: Raven PM47, Raven, 1986; handedness: Oldfield, 1971; see **Table 1**].

**Table 1:** Neuropsychological assessment.

	Raw score	Equivalent score or cut-off^∗^
**Neuropsychological tests**		
ROCF (copy accuracy)	13	>32^∗^
ROCF (recall accuracy)	4	>22^∗^
Corsi visuo-spatial span	<3	0
Single digit span	3	0
Trail making test – A	180″	Max 40″
Raven PM47	12	0
Attentional matrices	24	0
Token test	14,5	0
Denomination	8/10	
Oldfield	12	<6

*Extensive battery of neuropsychological tests was administered to the patient, that revealed a general global impairment of cognitive abilities (TMT, trail making test; ROCF, Rey–Osterrieth complex figure; Raven PM47, Raven’s colored progressive matrices PM47). Equivalent score and Cut-off score (^∗^) are the normalized raw score for age and gender data. (A score below the Cut-off is considered of clinical relevance and expression of a deficit in that function/domain; Equivalent scores go from 0 to 4, 0 is considered deficit, 1 = borderline, 2–3–4 are considered no deficit).*

Long-term video-electroencephalography (LT-VEEG) recording showed a frequent epileptic activity in bilateral temporal and frontal regions (single spike-and-waves complex and brief sequences of 2 Hz), with a unique seizure arising from the right frontal cortex (repetitive rhythmic spikes, maximum amplitude on Fp2, F4 and Fz; duration: 18 s; 10–20 EEG system), clinically manifested as a left head-turning and hyperextension of the thorax.

**FIGURE 1:**
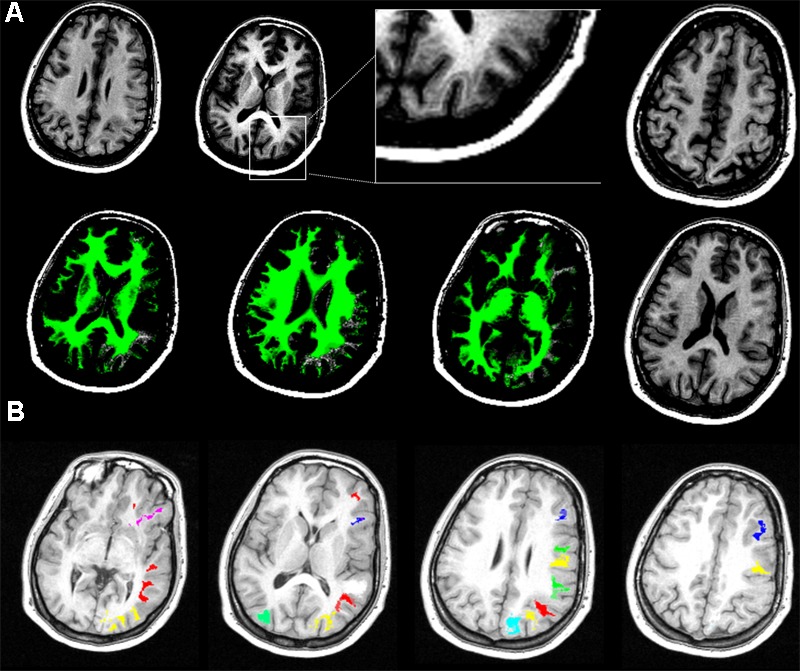
**(A)** Localization and extension of double-cortex (DC)-like heterotopias. T1-weighted and white matter mask (green) images show diffuse subcortical band heterotopias, simulating a “double cortex,” underneath the correctly migrated cortex. Images are displayed in neurological convention. **(B)** Laminar heterotopias’ regions of interest (ROI, color-coded). Double cortex was manually traced in order to create ROI (color-coded) related to the corresponding migrated anatomical regions (see section “Materials and Methods” for details).

Structural MRI imaging revealed diffuse subcortical band heterotopias, specifically located in the right prefrontal, temporal and bilateral occipital regions (**Figure 1A**). Identification and labeling of heterotopic bands was performed manually in order to: (i) characterize the connectivity profile of heterotopic and migrated gray matter, (ii) compare connectivity profile of heterotopic neurons and corresponding correctly migrated ones, and (iii) compare them with a sample of thirty age-matched (17 females, age 24.4 +/- 3.8 years) healthy controls.

**FIGURE 2:**
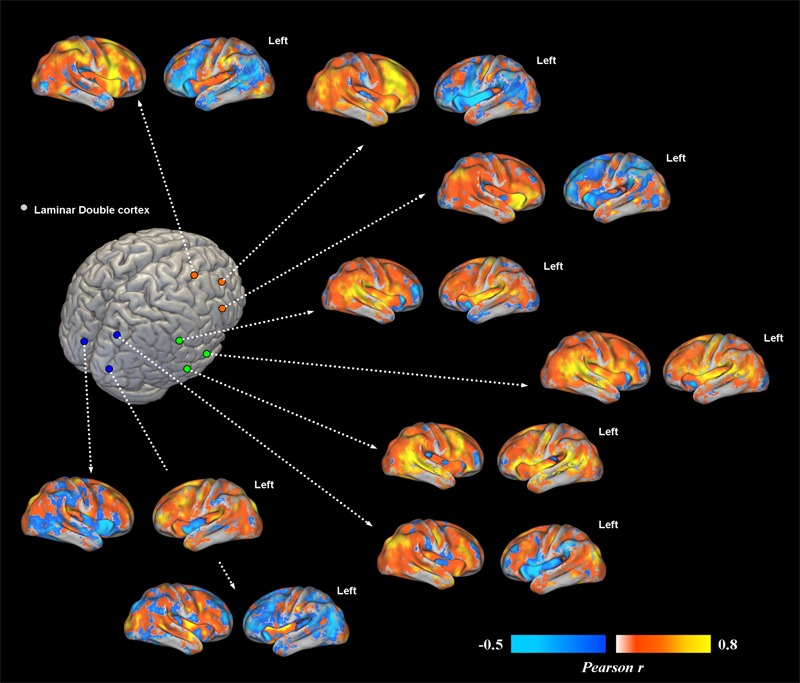
Voxel-wise functional connectivity of prefrontal, temporal and occipital heterotopias. Most representative areas show a stronger pattern of functional connectivity (FC) with their corresponding correctly migrated layers (i.e., high local connectivity, measured via Pearson correlation coefficient). Interhemispheric connections with the contralateral cortex are present only for ROIs placed in the temporal lobe.

**FIGURE 3:**
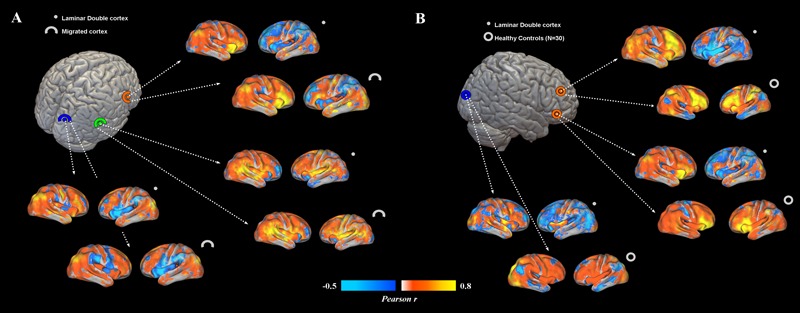
**(A)** Functional connectivity profiles of heterotopias and correctly migrated gray matter. Voxel-wise FC maps (with respect to properly migrated gray matter) are displayed for three exemplar brain locations representing prefrontal, temporal and occipital heterotopias, and corresponding migrated gray layers. Migrated gray matter shows a pattern of decreased interhemispheric connections similar to that of heterotopias, even if more interhemispheric connectivity is present. **(B)** FC profiles of heterotopias and healthy controls. FC of prefrontal and occipital heterotopias is reported and compared to that of 30 healthy controls. Reduced interhemispheric functional connections and lack of symmetry are clearly observable respect to of healthy subjects.

Overall, subcortical ROIs showed strong positive FC with their corresponding migrated gray matter (**Figure 2**). Interestingly, such strong ipsilateral connectivity pattern was more evident for prefrontal and bilateral occipital heterotopic regions, whereas right temporal lobe areas revealed a more balanced connectivity profile with both hemispheres (**Figure 2**). As for connectivity with the rest of the brain, correctly migrated gray matter showed a similar connectivity pattern to that of laminar subcortical gray matter, but a more pronounced connectivity with the contralateral hemisphere (**Figure 3A**). A difference between the FC profile of patient’s laminar gray matter and that of healthy controls was also evident, confirming a clear-cut reduction of inter-hemispheric connections (**Figure 3B**), with patterns of flipped connectivity – i.e., negative – present as well.

Genetic analysis revealed two variants in heterozygous state: c.739C > A (p.Pro247Thr) in FAT4 gene and c.4166G > A (p.Arg1389His) in COL18A1. Both variants affect autosomal recessive disease-causing genes and show a low frequency in the general population (MAF = 0,0038 for c.739C > A and MAF = 0,000008 for c.4166G > A, based on ExAC database). Segregation analyses indicated that the two variants were inherited from the mother and were absent in the healthy sister; father’s DNA was not available for the analysis. CGH analysis did not identify microscopically detectable chromosome rearrangements on the other allele of the two genes. A small duplication of approximately 101 Kb in 20p13, including PSMF1 gene, probably not pathogenic, was reported. Microscopically detectable copy-number variants (CNVs) have been excluded on the paternal allele. The study was approved by the Ethical Committee of the Le Scotte Hospital and University of Siena School of Medicine, in accordance with the Declaration of Helsinki. The patient’s family gave a written informed consent to the procedures as well as to anonymous publication of this case report.

### MRI Data Acquisition

Magnetic resonance imaging (MRI) data were acquired on a Philips Intera 1.5 T whole-body scanner. Resting-state fMRI data included 178 volumes with 33 axial slices covering the whole brain, acquired via a T2 blood oxygenation level dependent (BOLD) sensitive multi-slice echo planar imaging (EPI) sequence (TR/TE = 2.5 s/32 ms; field of view = 22 cm; image matrix = 64 × 64; voxel size = 3.44 mm × 3.44 mm × 3.8 mm; flip angle = 75°). Structural imaging was performed using a whole brain T1-weighted Fast Field Echo 1 mm^3^ sequence (TR/TE = 30/4.6 ms, field of view = 250 mm, matrix = 256 × 256, flip angle = 30°, slice number = 150). Participant was provided with earplugs. Particular care was taken to minimize head motion via vacuum cushions and custom-made padding.

### fMRI Preprocessing

Functional MRI (fMRI) data preprocessing and statistical analyses were carried out using SPM8 software (Statistical Parametric Mapping^1^) and MATLAB 7.5 (MathWorks, Natick, MA, United States). The first three volumes of functional images were discarded to allow for steady-state magnetization. EPI images were slice-time corrected using the interleaved descending acquisition criteria, and realigned and re-sliced to correct for head motion using a mean functional volume derived from the overall fMRI scans. Hidden Markov Random Field model was applied in all segmentation processes in order to remove isolated voxels. Customized tissue prior images and T1-weighted template were smoothed using an 8 mm full-width at half-maximum (FWHM) isotropic Gaussian kernel. Structural images were coregistered to functional images and subsequently segmented using the SPM unified segmentation routine. Linear trends were removed to reduce the influence of the rising temperature of the MRI scanner and all functional volumes were band pass filtered at (0.01 Hz < *f* < 0.08 Hz) to reduce low-frequency drift. Finally, a CompCor algorithm has been applied in order to control physiological high-frequency respiratory and cardiac noise.

### MRI Analysis

T1-weighted MRI image was segmented in grey and white matter tissue maps using non-linear approaches in SPM software; the white matter mask (green) was superimposed to the original T1 image and DC-like heterotopias were identified (right hemisphere = Brodmann Area -BA9, BA11, BA13, BA15, BA43, BA45, BA51, BA53, BA57, BA63, BA65, BA67, BA81, BA85, BA89; bilateral = BA50–52, **Figures 1A,B**). Two investigators independently classified each layer by size, visibility, band or nodular pattern, selecting nine representative ROIs encompassing occipital, temporal and prefrontal lobes (**Figure 1B**); a 3D volume including all the DC-like layers was created using MRIcron software. A map including BAs in MNI space was warped and co-registered to the patients MRI, superimposed to the DC-like volume, which was then separated in distinct ROIs according to the BA corresponding to each overlying, migrated cortical area. Functional connectivity (FC) was calculated by computing the Pearson product-moment correlation coefficient between the average BOLD time series extracted from each regions of interest (ROI) sampled at 1 mm^3^ resolution. Each of the ROIs was analyzed in a seed-to-voxel whole brain FC analysis using resting-state fMRI data co-registered to the structural MRI. ROIs of migrated gray matter layers corresponding to each heterotopia ROI were also created and included in the analysis, allowing the comparison of connectivity profile for migrated and heterotopic gray matter corresponding to the same BA. Finally, the connectivity of migrated gray matter ROIs was compared with that of the same regions in a sample of 30 age-matched healthy participants (17 females, age 24.4 +/- 3.8 years). Given the explorative case report nature of the present investigation, results refer to visual inspection of unthresholded FC maps.

### Genetic Analysis

For the genetic analysis, Next Generation Sequencing (NGS) and Sanger sequencing methods were adopted on a large dataset of genes associated with cortical malformations: ATP6V0A2, ACTB, ACTG1, ARFGEF2, B3GALNT2, B3GNT1, CNTNAP2, COL18A1, DCHS1, DCX, DYNC1H1, EML1, ERMARD, FAT4, FKRP, FKTN, FLNA, GMPPB, GPR56, GPSM2, ISPD, KIAA1279, KIF2A, KIF5C, LAMA2, LAMB1, LAMC3, LARGE, MEF2C, NDE1, NSDHL, OCLN, PAFAH1B1, PAX6, POMGNT1, POMGNT2, POMK, POMT1, POMT2, RAB18, RAB3GAP1, RAB3GAP2, RELN, RTTN, SNAP29, SRPX2, TBC1D20, TMEM5, TUBA1A, TUBA8, TUBB, TUBB2B, TUBB3, TUBG1, VLDLR, WDR62. In order to detect genomic rearrangements, array-based comparative genomic hybridization (array CGH) analysis was performed using Agilent Human Genome 244K Microarrays.

## Background

Periventricular neuronal Heterotopia (PH) is a heterogeneous congenital disorder of cortical development consisting in additional gray matter proximal to cerebral ventricular walls (Cappello et al., 2013), frequently determining drug-resistant seizures (Guerrini and Carrozzo, 2001). Nowadays, 15 subtypes of PH have been identified based on MRI findings and associated anomalies (Parrini et al., 2006), with many genes linked to the most frequent subtype (i.e., bilateral periventricular nodular heterotopia -PNH- to FLNA and ARFGEF2 genes, Parrini et al., 2006). Similarly, Double Cortex syndrome is a rare congenital malformation of cortical development consisting in an extra layer of neurons underneath the properly migrated cortex, mostly associated to Double Cortin X and Lissencephaly 1 genes’ mutation, usually causing drug-resistant epilepsy and severe cognitive deficits (Tanaka and Gleeson, 2008). Few cases of laminar PH displaying the MRI features of Double Cortex have been reported, without mutation of Double Cortin X (Cappello et al., 2013), opening a debate about the specific link between the development of “double-cortex”-like laminar PH and Double Cortin X gene. No data on the genetic mutation characterizing these patients are available to date, while only a handful of studies have investigated possible alteration of FC MRI patterns exclusively in patients with classical PNH (Archer et al., 2010; Christodoulou et al., 2012, 2013; Emiliano et al., 2012; Shafi et al., 2015) and task fMRI studies, showing an integration of DC in the normal sensorimotor circuits (Draganski et al., 2004; Keene et al., 2004; Jirsch et al., 2006; Briellmann et al., 2006).

We conducted for the first time a resting-state FC analysis and multi-gene analysis on a woman presenting a unique DC-like structural MRI profile. Considering prior evidence on PNH, we hypothesized that the patient would show positive FC between the extra gray matter layer and properly migrated neurons, as well as a decrement of interhemispheric connectivity, given the reduction of cortical population correctly migrated.

## Discussion

### Functional Connectivity

Functional connectivity analysis showed a functional involvement of laminar heterotopias in spontaneous, oscillatory cortical dynamics. The positive connectivity between laminar heterotopias and its corresponding migrated gray matter region matches previous evidence on DC syndrome obtained with task-fMRI investigations in which a simultaneously involvement with the corresponding overlying migrated cortex in sensorimotor and linguistic tasks was demonstrated (Draganski et al., 2004; Keene et al., 2004; Briellmann et al., 2006; Jirsch et al., 2006). Additionally, in PNH patients (Emiliano et al., 2012; Christodoulou et al., 2012) periventricular gray matter nodules showed synchronization with the overlying and surrounding cortex, as well as with the cerebellum bilaterally (Emiliano et al., 2012). However, in contrast with these previous data showing connectivity between heterotopic nodules and the contralateral hemisphere (Christodoulou et al., 2012), our patient displayed strongly reduced FC between laminar gray matter and the contralateral hemisphere compared to controls (**Figure 3B**). Such hemispheric segregation has not been reported in PNH and PH cases so far, possibly suggesting this as a specific feature of DC-like syndrome. Moreover, the positive FC with the overlying cortex suggests its possible inclusion into epileptogenic cortical circuits, as seems to emerge in the previous fMRI studies. For PNH, Christodoulou et al. (2013) investigated task-related fMRI activations during a reading task: they found synchronous activations of PNH with the correspondent ipsilateral overlying cortex, as well as with the contralateral ones. Interestingly, the pattern of co-activation during the task also reflected connectivity measured during resting-state. In the study of Shafi et al. (2015), a combined transcranial magnetic stimulation (TMS)-EEG investigation revealed an augmented late response [TMS-evoked potentials (TEPs)] in the stimulated cortical sites, previously selected on the basis of their positive connectivity with deeper heterotopias’ nodules. The authors postulated that PNH-related functional alterations have consequence on cortical physiology, particularly consisting in a reverberation of ictal activity in large-scale cortical circuits. As for DC syndrome, laminar heterotopia revealed a simultaneously BOLD activation with the overlying cortex (Tyvaert et al., 2008) during ictal and interictal events, even more evident for DC, leading the suggestion of its possible involvement in the generation of ictal and interictal events. Our long-term video-EEG data, revealing an ictal activity in the right hemisphere where laminar gray matter is located, are in line with this evidence. Additionally, the close to normal inter-hemispheric connectivity between right temporal heterotopic gray matter and the contralateral temporal lobe could reflect the persistence of an epileptogenic network as consequence of spikes spreading, as hypothesized by Shafi et al. (2015). Frequent seizures activity in reverberating circuitries can indeed modulate FC (Tracy and Doucet, 2015), therefore longitudinal functional neuroimaging evaluations of epileptic patients with DC-like (and DC) syndromes could help disentangle the causality between altered connectivity, band heterotopia and epileptic activity.

The reduced alteration of connectivity could also reflect patient’s cognitive deficits: patient showed moderate mental retardation with a global reduction of cognitive abilities without a prevalent impairment in linguistic and reading abilities as usually reported in PNH patients (Christodoulou et al., 2012). We could carefully suggest the more physiological connectivity between temporal lobes might explain her moderate impairment in language related functions, while, on the other hand, the profound alterations of interhemispheric connectivity might be one of the substrates for patient’s global cognitive deficit (**Table 1**).

### Genetic Analysis

Genetic analysis revealed negative findings on a large panel of known genes involved in cortical development, suggesting a not yet documented cortical malformation in our patient. The analysis highlighted two variants of maternal origin, involving FAT4 and COL18A1 genes. Biallelic mutations of FAT4 gene (FAT atypical cadherin) are associated with a rare autosomal recessive multiple malformation syndrome: the Van Maldergem syndrome (VMS), characterized by multiple bodily malformations (craniofacial, auditory, renal) and partially penetrant PH (Cappello et al., 2013). Even if a link between VMS and DC-like heterotopia has been proposed (Cappello et al., 2013), no data on the genetic determinant of this particular pattern are available to date. Interestingly, the typical distribution of heterotopias in the VMS syndrome involves the occipital horns. Accordingly, the laminar cortex in our patient is present bilaterally only in the occipital lobes. In addition, cadherins (codified by FAT4 genes) have an important role in the structural and left-right differentiation of brain, body (Petzoldt et al., 2012; Mendes et al., 2014), and language (Beste et al., 2016), and might be associated with the segregation of interhemispheric connectivity detected by fMRI analysis. Finally, Cappello et al. (2013) demonstrated that the FAT4-linked heterotopias are mostly due to an increase in neuroprogenitor cells number (i.e., with a block of their differentiation process), rather than to a pure alteration of mature neurons’ migration. This incomplete maturation of heterotopic neurons could even limit their ability to develop a balanced FC pattern, although no FC studies on neuroprogenitor cells are present to date. The other variant affects the COL18A1 gene, which is known to cause the Knobloch syndrome (severe vision impairments with occipital encephalocele), both features lacking in our patient (Spalice et al., 2009). Given the lack of availability of father’s DNA, it is not possible at this time to proceed with additional investigations.

Based on these findings, it is not possible to directly relate the malformation with the two mutations. However, we cannot completely rule out the possibility that the two variants play a role as cofactors of the condition, under the assumption of a multifactorial model. In fact, the phenotype of presentation is highly variable in epilepsy, also within patients with the same mutations, ranging from benign to severe disease (Weber et al., 2017). At the same time, it is not possible to exclude either an additional role of the CNV in 20p13 or a perturbative effect on the surrounding regions. The gene included in this location, PSMF1, plays an important role in the control of proteasome function, suggesting a possible role in neuronal function, even if mutations in this gene have not been correlated with specific human diseases.

### Diagnosis and Treatment

Considering video-EEG, MRI, genetic findings and the clinical presentation, a diagnosis of fronto-temporal focal epilepsy with secondary generalization caused by a double-cortex-like malformation was formulated, accordingly to the last ILAE Classification of the Epilepsies (Scheffer et al., 2017). The following pharmacological treatment was then assigned: Valproate 400 mg twice daily, Lamotrigine 150 mg twice daily, Clobazam 10 mg three times daily, Topiramate 150 mg twice daily. In the successive medical follow-up, patient did not report any collateral effects but ictal events (only simple motor partial seizures) persisted with a weekly frequency.

## Concluding Remarks

For the first time, connectivity and genetic analyses of an epileptic patient with unique case of DC-like heterotopia originally revealed: (i) strong FC between DC-like regions and their properly migrated gray matter counterparts, (ii) close to normal intra-hemispheric and reduced inter-hemispheric connectivity for each DC-like ROI (respect to the healthy controls), except for those located in the temporal lobe, as well as (iii) a similar decrease in contralateral connectivity for correctly migrated cortices with respect to age-matched healthy controls. Large panel of known genes involved in cortical development did not show any mutations, while two mutations in heterozygous state of recessive-diseases causing genes were detected, suggesting that our patient presented a new cortical malformation, not yet genetically assessed in literature. In fact, the two genes’ variants are extremely rare across the population, and so far, their combination was not revealed in any type of patient. To our knowledge, this is the first case report of a double-cortex-like patient extensively studied, in which we show a unique pattern of altered resting-state activity and a new association of genetic mutations. Considering the recent advances in understanding the implications of altered resting FC for the normal cognitive abilities (Sporns et al., 2000), intelligence (Finn et al., 2015), as well as neurological and psychiatric conditions (Sheffield and Barch, 2016; Drysdale et al., 2017), documenting altered FC in epileptic patients with cortical malformations could lead to a substantial improvement in understanding the neurophysiological/cognitive consequences and substrates of these complex conditions. Present data provide original evidence of altered interhemispheric FC dynamics and suggest that DC-like patients show separate genetic and neuroimaging findings compared to PNH and other form of PH.

## Author Contributions

ES, GV, and GS designed the study. ES, GV, AC, and GS acquired the MRI, EEG, and clinical data. ES conducted the MRI analysis. FZ, ARe, MM conducted the genetic analysis. GS, ES, GV, SR, FZ, ARe, MM, and ARo wrote the paper. SR and ARo supervised the study.

## Conflict of Interest Statement

The authors declare that the research was conducted in the absence of any commercial or financial relationships that could be construed as a potential conflict of interest.

## Footnotes

1 www.fil.ion.ucl.ac.uk/spm/

## References

[B1] Archer J. S. Abbott D. F. Masterton R. A. Palmer S. M. Jackson G. D. (2010). Functional MRI interactions between dysplastic nodules and overlying cortex in periventricular nodular heterotopia. *Epilepsy Behav.* 19 631–634. 10.1016/j.yebeh.2010.09.018 21030316

[B2] Beste C. Ocklenburg S. von der Hagen M. Di Donato N. (2016). Mammalian cadherins DCHS1-FAT4 affect functional cerebral architecture. *Brain Struct. Funct.* 221 2487–2491. 10.1007/s00429-015-1051-6 25930014

[B3] Briellmann R. S. Little T. Harvey A. S. Abbott D. F. Jacobs R. Waites A. B. (2006). Pathologic and physiologic function in the subcortical band of double cortex. *Neurology* 67 1090–1093. 10.1212/01.wnl.0000237554.39283.6b 17000988

[B4] Cappello S. Gray M. J. Badouel C. Lange S. Einsiedler M. Srour M. (2013). Mutations in genes encoding the cadherin receptor-ligand pair DCHS1 and FAT4 disrupt cerebral cortical development. *Nat. Genet.* 45 1300–1308. 10.1038/ng.2765 24056717

[B5] Christodoulou J. A. Barnard M. E. Del Tufo S. N. Katzir T. Whitfield-Gabrieli S. Gabrieli J. D. (2013). Integration of gray matter nodules into functional cortical circuits in periventricular heterotopia. *Epilepsy Behav.* 29 400–406. 10.1016/j.yebeh.2013.08.028 24090774 PMC3844926

[B6] Christodoulou J. A. Walker L. M. Del Tufo S. N. Katzir T. Gabrieli J. D. Whitfield-Gabrieli S. (2012). Abnormal structural and functional brain connectivity in gray matter heterotopia. *Epilepsia* 53 1024–1032. 10.1111/j.1528-1167.2012.03466.x 22524972 PMC3370071

[B7] Corrigan J. D. Hinkeldey N. S. (1987). Relationships between parts A and B of the Trail Making Test. *J. Clin. Psychol.* 43 402–409. 10.1002/1097-4679(198707)43:4<402::AID-JCLP2270430411>3.0.CO;2-E 3611374

[B8] De Renzi E. Vignolo L. (1962). The token test: a sensitive test to detect receptive disturbances in aphasics. *Brain* 85 665–678. 10.1093/brain/85.4.665 14026018

[B9] Draganski B. Winkler J. Flugel D. May A. (2004). Selective activation of ectopic grey matter during motor task. *Neuroreport* 15 251–253. 15076746 10.1097/00001756-200402090-00007

[B10] Drysdale A. T. Grosenick L. Downar J. Dunlop K. Mansouri F. Meng Y. (2017). Resting-state connectivity biomarkers define neurophysiological subtypes of depression. *Nat. Med.* 23 28–38. 10.1038/nm.4246 27918562 PMC5624035

[B11] Emiliano S. Giampaolo V. Daniela M. Nicola P. Alfonso C. Raffaele R. (2012). Cerebro-cerebellar functional connectivity profile of an epilepsy patient with periventricular nodular heterotopia. *Epilepsy Res.* 101 280–283. 10.1016/j.eplepsyres.2012.04.006 22542195

[B12] Finn E. S. Shen X. Scheinost D. Rosenberg M. D. Huang J. Chun M. M. (2015). Functional connectome fingerprinting: identifying individuals using patterns of brain connectivity. *Nat. Neurosci.* 18 1664–1671. 10.1038/nn.4135 26457551 PMC5008686

[B13] Guerrini R. Carrozzo R. (2001). Epileptogenic brain malformations: clinical presentation, malformative patterns and indications for genetic testing. *Seizure* 10 532–543. 10.1053/seiz.2001.0650 11749114

[B14] Jirsch J. D. Bernasconi N. Villani F. Vitali P. Avanzini G. Bernasconi A. (2006). Sensorimotor organization in double cortex syndrome. *Hum. Brain Mapp.* 27 535–543. 10.1002/hbm.20197 16124015 PMC6871446

[B15] Kaplan E. Goodglass H. Weintraub S. (1983). *The Boston Naming Test.* Philadelphia, PA: Lea and Febiger.

[B16] Keene D. L. Olds J. Logan W. J. (2004). Functional MRI study of verbal fluency in a patient with subcortical laminar heterotopia. *Can. J. Neurol. Sci.* 31 261–264. 10.1017/S0317167100053920 15198455

[B17] Mendes R. V. Martins G. G. Cristovao A. M. Saude L. (2014). N-cadherin locks left-right asymmetry by ending the leftward movement of Hensen’s node cells. *Dev. Cell* 30 353–360. 10.1016/j.devcel.2014.06.010 25117685

[B18] Oldfield R. C. (1971). The assessment and analysis of handedness: the Edinburgh inventory. *Neuropsychologia* 9 97–113. 10.1016/0028-3932(71)90067-4 5146491

[B19] Orsini A. (1993). *Contributo Alla Taratura Italiana Della WISC-R, O S.* Firenze: Organizzazioni Speciali.

[B20] Osterrieth P. A. (1944). Le test de copie d’une figure complex: contribution a l’etude de la perception et de la memoire. *Arch. Psychol.* 30 286–356.

[B21] Parrini E. Ramazzotti A. Dobyns W. B. Mei D. Moro F. Veggiotti P. (2006). Periventricular heterotopia: phenotypic heterogeneity and correlation with Filamin A mutations. *Brain* 129 1892–1906. 10.1093/brain/awl125 16684786

[B22] Petzoldt A. G. Coutelis J. B. Geminard C. Speder P. Suzanne M. Cerezo D. (2012). DE-Cadherin regulates unconventional Myosin ID and Myosin IC in *Drosophila* left-right asymmetry establishment. *Development* 139 1874–1884. 10.1242/dev.047589 22491943

[B23] Raven J. C. (1986). *Manual for Raven’s Progressive Matrices and Vocabulary Scales (Section 2).* London: Lewis.

[B24] Scheffer I. E. Berkovic S. Capovilla G. Connolly M. B. French J. Guilhoto L. (2017). ILAE classification of the epilepsies: position paper of the ILAE commission for classification and terminology. *Epilepsia* 58 512–521. 10.1111/epi.13709 28276062 PMC5386840

[B25] Schoenberg M. R. Dawson K. A. Duff K. Patton D. Scott J. G. Adams R. L. (2006). Test performance and classification statistics for the Rey Auditory Verbal Learning Test in selected clinical samples. *Arch. Clin. Neuropsychol.* 21 693–703. 10.1016/j.acn.2006.06.010 16987634

[B26] Shafi M. M. Vernet M. Klooster D. Chu C. J. Boric K. Barnard M. E. (2015). Physiological consequences of abnormal connectivity in a developmental epilepsy. *Ann. Neurol.* 77 487–503. 10.1002/ana.24343 25858773 PMC4394240

[B27] Sheffield J. M. Barch D. M. (2016). Cognition and resting-state functional connectivity in schizophrenia. *Neurosci. Biobehav. Rev.* 61 108–120. 10.1016/j.neubiorev.2015.12.007 26698018 PMC4731300

[B28] Spalice A. Parisi P. Nicita F. Pizzardi G. Del B. F. Iannetti P. (2009). Neuronal migration disorders: clinical, neuroradiologic and genetics aspects. *Acta Paediatr.* 98 421–433. 10.1111/j.1651-2227.2008.01160.x 19120042

[B29] Sporns O. Tononi G. Edelman G. M. (2000). Connectivity and complexity: the relationship between neuroanatomy and brain dynamics. *Neural Netw.* 13 909–922. 10.1016/S0893-6080(00)00053-8 11156201

[B30] Tanaka T. Gleeson J. G. (2008). Subcortical laminar (band) heterotopia. *Handb. Clin. Neurol.* 87 191–204. 10.1016/S0072-9752(07)87012-6 18809026

[B31] Tracy J. I. Doucet G. E. (2015). Resting-state functional connectivity in epilepsy: growing relevance for clinical decision making. *Curr. Opin. Neurol.* 28 158–165. 10.1097/WCO.0000000000000178 25734954

[B32] Tyvaert L. Hawco C. Kobayashi E. LeVan P. Dubeau F. Gotman J. (2008). Different structures involved during ictal and interictal epileptic activity in malformations of cortical development: an EEG-fMRI study. *Brain* 131 2042–2060. 10.1093/brain/awn145 18669486 PMC3792088

[B33] Weber Y. G. Biskup S. Helbig K. L. Von Spiczak S. Lerche H. (2017). The role of genetic testing in epilepsy diagnosis and management. *Expert Rev. Mol. Diagn.* 17 739–750. 10.1080/14737159.2017.1335598 28548558

